# Smart devices to measure and monitor QT intervals

**DOI:** 10.3389/fcvm.2023.1172666

**Published:** 2023-11-27

**Authors:** Leendert J. Hoek, Jan Leendert P. Brouwer, Adriaan A. Voors, Alexander H. Maass

**Affiliations:** ^1^ ICON plc, Early Development Services, Groningen, Netherlands; ^2^ Department of Cardiology, University of Groningen, University Medical Center Groningen, Groningen, Netherlands

**Keywords:** QTc, QT interval, smartwatch, smart device, ECG

## Abstract

Careful observation of the QT interval is important to monitor patients with long QT syndrome and during treatment with potentially QT-prolonging medication. It is also crucial in the development of novel drugs, in particular in case of a potential side effect of QT prolongation and in patients with increased risk of QT prolongation. The 12-lead electrocardiogram (ECG) is the gold standard to evaluate cardiac conduction and repolarization times. Smartwatches and smart devices offer possibilities for ambulatory ECG recording and therefore measuring and monitoring the QT interval. We performed a systematic review of studies on smartwatches and smart devices for QTc analysis. We reviewed PubMed for smartwatches and smart devices that can measure and monitor the QT interval. A total of 31 studies were included. The most frequent devices were (1) KardiaMobile 6L, a Food and Drug Administration-approved device for QTc analyses that provides a 6-lead ECG, (2) an Apple Watch, a smartwatch with an integrated ECG tool that allows recording of a single-lead ECG, and (3) the Withings Move ECG ScanWatch, an analog watch with a built-in single-lead ECG. The KardiaMobile 6L device and the Apple Watch provide accurate measurements of the QT interval, although the Apple Watch is studied in standard and non-standard positions, and the accuracy of QT measurements increased when the smartwatch was moved to alternative positions. Most studies were performed on patients, and limited results were available from healthy volunteers.

## Introduction

In 1957, Jervell and Lange-Nielsen described a case of a family in which QT prolongation was found in multiple children and who subsequently died in infancy without any evidence of cardiac pathology at autopsy (1). Descriptions of young individuals with prolonged QT intervals and a history of loss of consciousness and ventricular fibrillation were published in the following years (2, 3). As a result, physicians showed increased awareness and recognized the importance of QT interval evaluation, acknowledging that abnormal QT prolongation may predispose to ventricular arrhythmia and sudden cardiac death. In 1964, Selzer and Wray described cases of ventricular tachycardia in the context of a prolonged QT interval in patients prescribed with Quinidine (4). The typical morphology of ventricular tachycardia was coined Torsades de Pointes (TdP) by Dessertenne (5). Congenital long QT syndrome (LQTS) is a familial cardiac ion channelopathy. Incomplete penetrance and variability in genetic expression lead to a heterogeneous phenotype. Classifying this condition clinically can be challenging (6). Those patients requiring regular QT interval monitoring are the mutation carriers, especially at a younger age. An increase in the QT interval can have therapeutic consequences, such as drug treatment with beta-blockers or pacemaker implantation. The diagnosis of LQTS partly depends on the QT interval, at rest or during recovery from the exercise stress test. Furthermore, T-wave morphology and clinical and family history are a part of the Scoring System for Clinical Diagnosis of Long QT Syndrome (7). In 1988, it was found that Prenylamine (Segontin) was associated with QT prolongation and sudden cardiac death. This resulted in Prenylamine being the first drug to be withdrawn from the market due to QT prolongation associated with sudden cardiac death (8). Additional classes of medications were linked to ventricular arrhythmias and cardiac death in the following years. Some of these agents were thereafter withdrawn by the Food and Drug Administration (FDA) (9). Due to these events, the pharmaceutical industry and government regulators became aware that careful evaluation of the QT interval during the development of a new compound devolvement program is crucial. There are still drugs on the market that have been associated with prolongation of the QT interval, such as patients with a need for psychotropic medications, and are linked with lethal ventricular arrhythmias (10). Monitoring the QT interval in patients prescribed this kind of medication could be of additional value. The ICH E14 guidance for industry mentions that other ways of obtaining a high-quality ECG can be used to collect ECGs for QT/QTc collection (11, 12). The gold standard for evaluating cardiac conduction and repolarization times is the 12-lead electrocardiogram (ECG), which is usually registered for seconds or minutes. For longer monitoring, Holter analysis can provide QT analysis for several days. The disadvantage of using a 12-lead ECG is that this also entails practical difficulties, including that the 12-lead ECG is just a single time point recording. Continuous monitoring is of added value in some situations. That way, patients can be monitored at home and possible QT prolongation after medication with possible effects on the QT time can be objectified more safely and easily. Other technologies have been developed to measure conduction times, including the QT interval. The reliability of these different devices is actively being investigated. The European Heart Rhythm Association has published a position paper on using digital devices to detect and manage arrhythmias (13). They conclude that for QT interval monitoring, studies are scarce and more studies are needed before these devices can be safely used on patients. Previous reviews on ECG monitoring systems were performed in the era before ECG recordings could be performed with smartwatches and therefore did not include QTc monitoring using these devices (14, 15). Other reviews on the use of smartwatches were related to detecting atrial fibrillation (16). To the best of our knowledge, this is the first review on using smartwatches to monitor QT intervals. This systematic review of the literature about the use of smartwatches and smart devices for QTc analysis is intended to provide an overview of the current literature regarding the use of these devices in analyzing QT intervals and to explore how these devices could change the landscape of QTc analysis.

## Materials and methods

We reviewed PubMed (https://pubmed.ncbi.nlm.nih.gov) for studies published on the use of smart devices for QTc analysis until September 30, 2022. For reporting and methodology, the updated 2020 Preferred Reporting Items for Systematic Reviews and Meta-Analysis guidelines were used (17). Terms “QTc” and “smart device,” “QT interval” and “smart device,” “QTc” and “smartwatch,” “QT interval” and “smartwatch,” “QTc” and “Apple Watch,” “QT interval” and “Apple watch,” “QTc” and “device,” “QT interval” and “device,” “device” and “TQT,” and “smartwatch” and “TQT” were used to identify studies examining the use of smart devices for QT analysis. Bibliographies of selected articles were manually reviewed for additional studies. Only original research articles published in English were considered for review. Eligibility of the articles was determined based on the screening of titles and abstracts. Articles that did not publish about methods and/or devices for QT analysis, implantable devices, 12-lead ECG monitoring, bed-side ECG monitoring; pediatric studies; non-human studies; and studies about telemetric monitoring were excluded.

## Results

**Figure 1:**
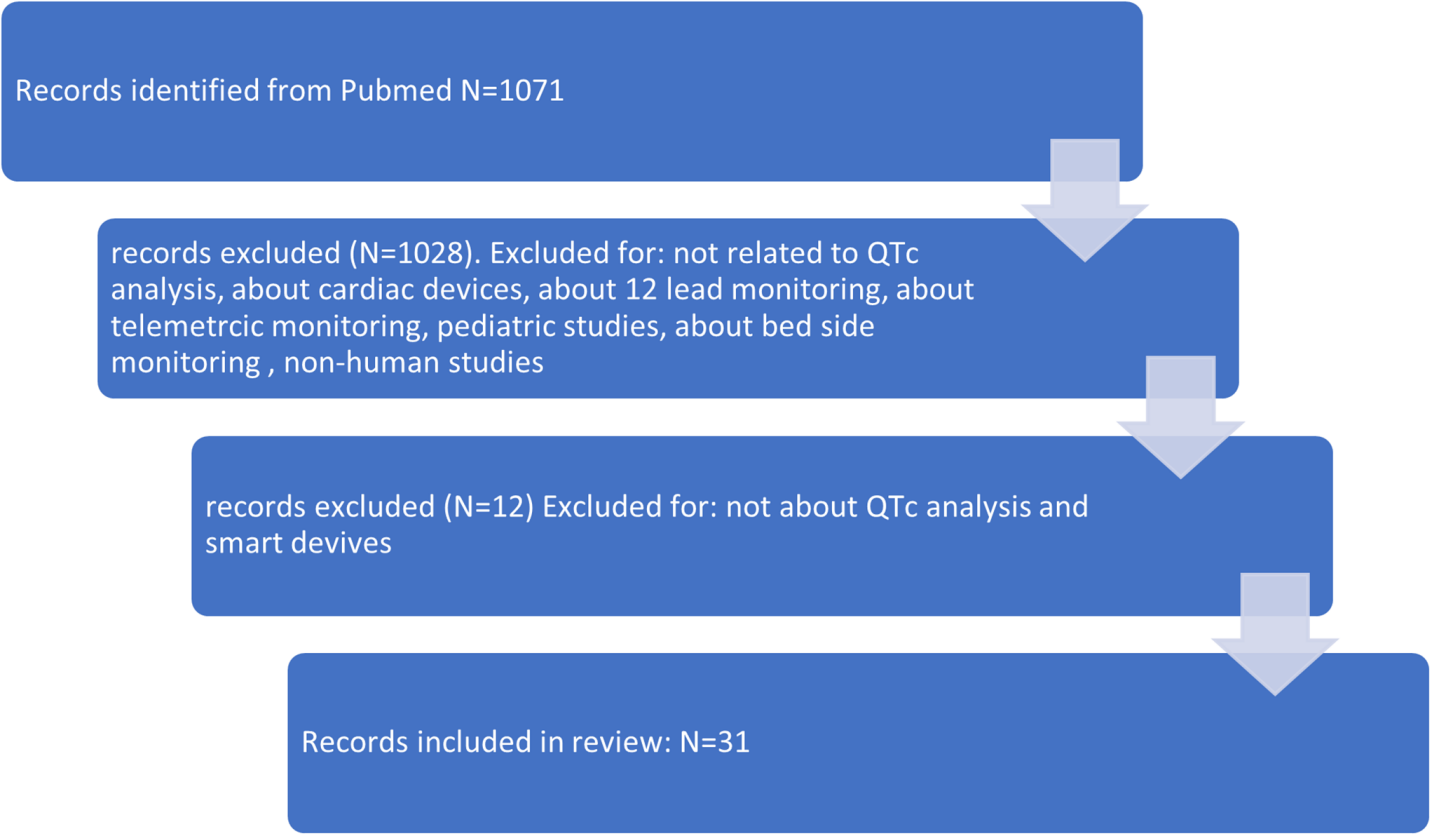
Search strategy.

**Figure 2:**
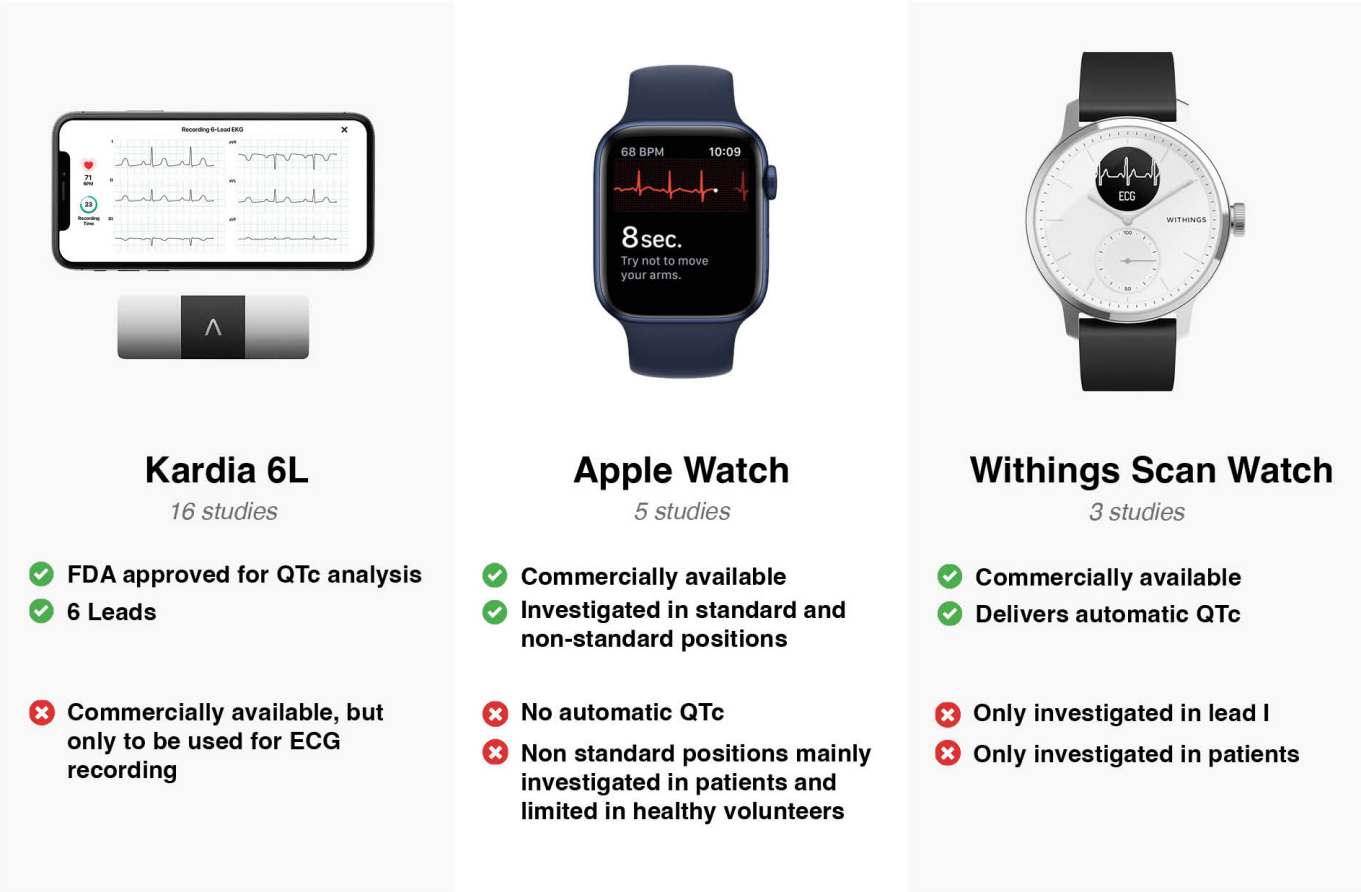
Graphical representation of most studied devices.

The initial search identified 1,071 studies. After screening titles, 43 articles were considered for further review. After reviewing the 43 articles, 12 articles were further excluded. The search strategy is shown in Figure 1. The search identified studies conducted until September 2022. The most frequently studied device was AliveCor's KardiaMobile (*N* = 16). Five studies examined the Apple Watch. Another smart watch (SW), the Withings Move ECG ScanWatch, was examined in three studies. A graphic representation of the three most studied devices is shown in Figure 2. In addition to the above-mentioned devices, a single publication was found for eight other devices an overview of the studied devices is shown in Table 1. Agreement between devices and 12-lead ECG was performed through Bland–Altman analysis in several studies and in a descriptive manner in some other publications.

**Table 1:** Table with an overview of studied devices.

KardiaMobile 6L						
Aim	*N*	Setting	Device	Leads	Outcome	Reference
Comparison of interval duration measurements between standard 12 lead ECGs and 6 Lead ECGs recorded with KardiaMobile 6L	705	Patients referred to the Genetic Heart Rhythm Clinic	KardiaMobile	ECG leads I, II, III, aVL, aVR, and aVF	Mean difference between the QTc measured on the 6-lead and 12-lead ECGs was −2.6 ms (95% CI −4.1 to −1.1 ms)	(20)
To access the accuracy of KardiaMobile 6L in measuring the QTc	234	Patients visiting the cardiology clinic for any indication	KardiaMobile	ECG leads I, II, III, aVL, aVR, and aVF	Mean absolute difference in QTc values between the modalities using lead I was 14 ± 13 ms (*r* = 0.783; <0.001). Mean absolute difference in lead II QTc between the modalities was 12 ± 9 ms (*r* = 0.856, *p* < 0.001)	(18)
To access the feasibility of obtaining recordings using the KardiaMobile 6L and to qualitative compare with standard 12-lead ECG recordings	4	COVID-19-positive patients or patients requiring ECG monitoring	KardiaMobile	ECG leads I, II, III, aVL, aVR, and aVF	KardiaMobile 6L had the ability to provide contactless ECGs with acceptable QT/QTc interval measurements	(21)
To describe the usefulness of telemonitoring for management of QT-prolonging drugs	70	COVID-19-positive patients receiving hydroxychloroquine, azithromycin, or lopinavir/ritonavir	KardiaMobile	ECG leads I, II, III, aVL, aVR, and aVF	Intraclass correlation coefficient points to a good agreement in the measurements of QTc interval	(22)
To investigate the KardiaMobile 6L to record and measure the QTc	13	Multidrug-resistant tuberculosis and non-tuberculous mycobacterium	KardiaMobile	ECG leads I, II, III, aVL, aVR, and aVF	Mean percentage difference between the automated 12-lead and manually calculated AliveCor readings was 3%. The correlation between the automated QTc and AliveCor QTc was evaluated with Pearson's correlation coefficient = 0.43 (*p* > 0.05)	(23)
To evaluate the agreement and clinical precision of Kardia Mobile 1l to measure the QTc interval and compare it to the 12-lead ECG	128	Patients with a presumed or confirmed diagnosis of COVID-19	KardiaMobile 1l	Single-lead ECG	Values of the QTc interval were practically the same for both devices (442.45 ± −40.5 vs. 441.65 ± 40.3 ms, *p *=* *5.15)	(24)
To evaluate the feasibility of QTc monitoring with a KardiaMobile 6L	227	182 patients with COVID-19 and 45 healthy patients	KardiaMobile 6L	ECG leads I, II, III, aVL, aVR, and aVF	No differences were observed between the monitoring strategies in QTc prolongation (*p* = 0.864). In the control group, all but one ECG registry with the smart device allowed QTc measurement, and mean QTc did not differ between both techniques (*p* = 0.612), displaying a moderate reliability [ICC 0.56 (0.19–0.76)]	(25)
To access the reliability of using AliveCor tracings (KardiaMobile 1l) and compare them to the QTc on standard ECGs	5	Patients on dofetilide for atrial fibrillation	KardiaMobile	Single-lead ECG	No significant difference between the AliveCor QTc and ECG QTc for any of the five patients (all ± 20 ms)	(26)
To determine the accuracy of different ECG-based devices to detect atrial fibrillation, QRS morphology, and ECG intervals compared with 12-lead ECG	176	Patients with congenital heart disease	KardiaMobile	ECG leads I, II, III, aVL, aVR, and aVF	QTc duration accuracy was acceptable in 74% of KardiaMobile 6L. QTc interval of KardiaMobile 6L compared to the 12-lead ECG illustrates limits of agreements, which were independent of the QTc interval	(27)
To train and validate an artificial intelligence-enabled 12-lead algorithm to determine the QTc and test this algorithm on tracings acquired from a KardiaMobile 6L	686	Patients with genetic heart disease	KardiaMobile	ECG leads I, II, III, aVL, aVR, and aVF	Difference between DNN-predicted QTc values derived from mECG tracings and those annotated from 12-lead ECGs by a QT expert (−0.45 ± 24.73 ms) and a commercial core ECG laboratory (10.52 ± 25.64 ms) was nominal	(33)
To describe the implementation of a remote trial in which self-collected ECG measurements were recorded on KardiaMobile 6L	231	Patients with SARS-CoV-2	KardiaMobile	ECG leads I, II, III, aVL, aVR, and aVF	QT interval can be efﬁciently measured and veriﬁed within a remote clinical trial paradigm	(34)
To compare the KardiaMobile 6L with the 12-lead ECG	1,015	Unselected cardiac inpatients and outpatients	KardiaMobile	ECG leads I, II, III, aVL, aVR, and aVF	Mean differences between KardiaMobile 6L and the 12-lead ECG for QT and QTc were small; the AUC was >75% for QT but less for QTc, although overall >60%	(28)
To examine and compare the level of similarity between KardiaMobile 6L ECG and 12-lead ECG	30	Healthy athletes	KardiaMobile	ECG leads I, II, III, aVL, aVR, and aVF	Relatively high levels of agreement between the mean 6-lead and 12-lead measurements for QTc, with the 6l readings slightly but significantly shorter on average. The difference in the QTc intervals was 391 vs. 401 ms (*p* = 0.003)	(29)
Comparison of KardiaMobile 6L and 12-lead ECG recordings	100	Cardiac patients	KardiaMobile	ECG leads I, II, III, aVL, aVR, and aVF	QT intervals measured by the KardiaMobile device were significantly different (shorter) than those observed in the standard ECG method: 393 vs. 400 ms (*p* < 0.001)	(30)
To determine the accuracy of QT measurement in a KardiaMobile 1l and compared it with a 12-lead ECG	125	Patients with non-acute indication in primary care	KardiaMobile	Single-lead ECG	Mean QTcB interval was 393 ± 25 ms in 1-lead ECGs and 392 ± 27 ms in lead I of the 12-lead ECGs, with a mean difference of 1 ± 21 ms. Comparing QTcB of 1-lead ECGs with those of lead II of 12-lead ECGs showed a mean difference of 8 ± 22 ms	(31)
To provide a brief overview of a protocol for monitoring the QT interval using KardiaMobile 6L	81	Patients with SARS-CoV-2	KardiaMobile	ECG leads I, II, III, aVL, aVR, and aVF	Portable wireless devices may represent a quick and useful alternative for QT interval monitoring	(32)
Apple Watch						
To compare the feasibility and reliability using the Apple Watch to calculate a QT interval to those of using a standard ECG to calculate a QT interval	119	100 patients admitted to Cardiology division 19 healthy subjects	Apple Watch	Leads I, II, and V2	There was agreement among the QT intervals of I, II, and V2 leads and the QT mean using the smartwatch and the standard ECG with Spearman's correlations of 0.886, 0.881, 0.793, and 0.914 (*p* < 0.001), respectively	(38)
To access the accuracy of interval measurements on Apple Watch tracings in comparison to lead I on a 12-lead ECG	43	Healthy volunteers	Apple Watch	Lead I of Apple Watch and lead I of 12-lead ECG	Mean difference (*d*) of –11.27 ± 22.9 ms for the QT interval (*r* = 0.79) and –11.67 ± 27 ms for the QTc interval (*r* = 0.57)	(39)
To compare the smartwatch-recorded QT and QTc assessed using AccurKardia's AccurBeat platform with the 12-lead ECG	50	Healthy volunteers	Apple Watch	Lead I Apple Watch and ECG leads I and II of 12-lead ECG	The Bland–Altman plot results found that 96% of the average QTc interval measurements between the platform and QTc intervals from the 12-lead ECG were within the 95% confidence limit of the average difference between the two measurements, with a mean difference of –10.5 (95% LoA −71.43 to 50.43). A total of 94% of the average QT interval measurements between the platform and the 12-lead ECG were within the 95% CI of the average difference between the two measurements, with a mean difference of –6.3 (95% LoA −54.54 to 41.94)	(40)
To validate the use of the Apple Watch for QT measurement	100	100 patients in sinus rhythm from outpatient or emergency departments	Apple Watch	Apple Watch lead I, lead II, and AW-LAT (simulated lead V6)	Compared with the 12-lead ECG, the median absolute error in QTc was 18 ms for AW-I, 20 ms for AW-II, and 16 ms for AW-LAT	(41)
To demonstrate the use of an Apple Watch to monitor QT prolongation	1	One patient with COVID-19	Apple Watch	Apple Watch lead I	Very similar waveform morphology and QT measurements compared to lead I of the 12-lead ECG	(42)
Withings ScanWatch						
To compare automated QTc measurements using a single-lead ECG of a Withings ScanWatch with manual measured QTc from a 12-lead ECG	367	Patients referred to a tertiary hospital for cardiac work-up	Withings ScanWatch	Smartwatch lead I	Disagreement for QTc measurements between the SW-AI and the manual measurements by the cardiologist using the 12-lead ECG was <15 ms in 38% cases and >20 ms in 54, and 29% of measurements had a disagreement >30 ms. In 12 patients (7%), the difference between the QTc intervals was greater than the LoA	(43)
To compare QTc duration measured on Withings ScanWatch compared with those measured on 12-lead ECGs	85	Patients with COVID-19 who were prescribed hydroxychloroquine-azithromycin therapy	Withings ScanWatch	Smartwatch lead I	Bland–Altman analysis resulted in a bias of 6.6 ms (95% LoA −59 to 72 ms) comparing automated QTc measurements (SW-ECG) with manual QTc measurement (12-lead ECG). In 12 patients (6.9%) the difference between the two measurements was greater than the LoA	(44)
To determine the accuracy of different ECG-based devices to detect atrial fibrillation, QRS morphology, and ECG intervals compared with 12-lead ECG	176	Patients with congenital heart disease	Withings ScanWatch	Smartwatch lead I	In the Withings ECG, the QTc interval was more frequently (49%) over- or underestimated by more than 40 ms compared to both Eko DUO (30%) and KardiaMobile 6L (26%) (*p* < 0.001 for both comparisons)	(27)
Other devices						
To evaluate the diagnostic accuracy of a patient-operated ECG device compared with a 12-lead ECG	508	Patients with an indication for 12-lead ECG recording	Omron HeartScan	Single-lead, position chest electrode C4	Linear correlation (*r*^2^) between the patient-operated ECG system and the standard ECG was 0.89 for QTc	(45)
To evaluate the ease of device use and quality of transmitted ECG tracings for QT interval measurement	31	Adult heart transplant recipients	HeartOne, Aerotel medical systems	Lead II	89% of the ECGs were acceptable quality for QT interval measurement	(46, 47)
To access the diagnostic accuracy of a single-lead portable ECG device for measuring QT intervals in comparison with a 12-lead ECG	101	Adult patients visiting the outpatient department with an indication for a 12-lead ECG recording	HeartCheck	Single-lead portable ECG Lead I	The mean QTc interval measured was 430.6 (SD ± 31.1) ms for the 12-lead ECG and 396.7 (SD ± 47.5) ms for the single-lead ECG. The difference of the QTc intervals between the two measurements was substantially outside the definition of perfect agreement of 10 ms difference or less. Only seven (6.9%) ECG recordings demonstrated perfect agreement	(47)
To evaluate ECG signal quality and ECG parameters measured with a 12-lead ECG acquisition T-shirt	30	Healthy subjects	12-lead ECG acquisition shirt	12 leads	QTc intervals obtained with the smart T-shirt were highly comparable to the ones measured with Holter	(48)
To evaluate the accuracy of a Smartphone Home Monitor for assessing the QTc as compared to the 12-lead ECG	124	99 healthy volunteers and 25 hospitalized patients receiving sotalol or dofelitide	Smartphone heart monitor	Leads I and II	In healthy volunteers the ASHM QT demonstrated a very good agreement (bias = 4 ms; standard deviation of bias = 11 ms) with the GE 12-lead ECG, using the Bland–Altman method of measurement agreement. In the hospitalized patients, the automated GE and ASHM QTc measurements based on lead I demonstrated a reasonable agreement (bias = 3 ms; standard deviation of bias = 46 ms) using the Bland–Altman method	(49)
To explore whether automated QTc measurements by BodyGuardian are sufficiently reliable compared to manual measurements on 12-lead Holter recordings	36	20 LQTS patients and 16 healthy controls	BodyGuardian	Lead II	QTc automatically measured by BG was 445 ± 47 ms, and the QTc manually measured was 446 ± 41 ms. The disagreement between BG and manual measurement was <15 ms in 57% of cases 34% of measurements had a disagreement >20 ms	(50)
To evaluate the diagnostic accuracy of a handheld bipolar ECG event recorder	52	52 patients admitted to the cardiology department	Beurer ME 80 device	Reconstruct 9 leads I, II, III, and V1–V6	Diagnosis of a prolonged QTc was inaccurate due to the inherent difficulties with measuring this interval because of lower signal quality and non-simultaneous tracings that make it difficult to align the waveforms	(51)
To evaluate the accuracy, usability, and diagnostic capabilities of a single-lead ECG device	144	94 patients cardiac patients and 50 asymptomatic controls	ECG check	Lead I	No significant differences were found in QT intervals between the two modalities	(52)

DNN, deep neural network; AW, apple watch; LAT, lateral; ICC, intraclass correlation.

### KardiaMobile 6L

KardiaMobile 6L (AliveCor Inc., Mountain View, CA, USA) is a wireless mobile ECG (mECG) device that can directly record a 6-lead ECG, which consists of leads I, II, and III and also augmented Vector Left (aVL), augmented Vector Foot (aVF), and augmented unipolar right arm lead (aVR). It is a small (9.0 cm × 3.0 cm × 0.72 cm) device that consists of three electrodes each on both the top surface and the bottom surface. Electrodes on the top surface make contact with both thumbs, and electrodes on the bottom surface make contact with either the left knee or the left ankle. KardiaMobile 6L can subsequently be connected to the corresponding application through Bluetooth on mobile devices such as tablets and smartphones to record a 30-s 6-lead mECG. It then provides an automated assessment of heart rate and heart rhythm (18). The FDA guidance allows using KardiaMobile 6L to measure QT intervals in patients with COVID-19 (19). Sixteen studies examined AliveCor's KardiaMobile 6L. Kleiman et al. (20) compared interval duration measurements (IDMs) between 6-lead ECGs recorded with AliveCor's KardiaMobile 6L and standard 12-lead ECGs. Interpretable 12-lead and 6-lead recordings were available for 685 out of 705 (97%) eligible patients. The mean difference between the QTc measured on the 6-lead and 12-lead ECGs was −2.6 ms (95% CI −4.1 to −1.1 ms). The absolute difference of <10 ms was present in 44.3%, ≤10 and <20 ms in 32.9%, ≤20 and <30 ms in 10.3%, ≤30 and <40 ms in 7.5%, ≤40 and <50 ms in 2.8%, and ≥50 ms in 2.2%. The authors concluded that 6-lead recordings with this KardiaMobile 6L can provide high-quality ECG recordings that may be useful in clinical medicine and during clinical trials. Bergeman et al. (18) studied the accuracy of the KardiaMobile 6L device for assessment of QT intervals in 234 outpatients visiting a cardiology clinic for any indication. Due to artifacts, it was impossible to perform QTc measurement in any lead in 16 mECGs (7%). In all 12-lead ECGs, QTc measurement was possible. Lead II was the most accurate lead. The mean (±SD) absolute difference in QTc values between mECGs and 12-lead ECGs was 12 ± 9 ms (*r* = 0.856; *p* < 0.001) in lead II. The absolute difference between QTc values was <10 ms in 55% of the subjects. A mean QTc ≥480 ms in lead II on the 12-lead ECG was found in six subjects. The sensitivity and speciﬁcity for mECG QTc prolongation in lead II were 80% and 99%, respectively (*n* = 203). The authors concluded that using a 6-lead mECG enables measuring the QT interval with good accuracy compared with the standard 12-lead ECG. Frisch et al. (21) published a case series of four patients in which they assessed the feasibility of obtaining mECG recordings using the KardiaMobile 6L device. Acceptable QT/QTc interval measurements were performed. Abellas-Sequeiros et al. (22) published a research letter about QT interval monitoring in patients with COVID-19 with KardiaMobile 6L. Seventy patients were enrolled, and tracings obtained with KardiaMobile 6L were of sufficient quality to provide an accurate QT interval measurement in 69 of them (98.6%). The device proved useful for ECG monitoring in these patients, detecting ECG abnormalities significant enough to promote a change in treatment in 17.4% of them. Puranik et al. (23) investigated the AliveCor device to monitor the QT interval in patients with multidrug-resistant tuberculosis and non-tuberculous mycobacterium. For 13 patients, a comparison was made between an automated QTc readout from the 12-lead ECG, and the mean QTc value was calculated from each patient's respective AliveCor device tracing (lead II). The AliveCor device underestimated the QTc compared to the corresponding 12-lead QTc readout in 12 of 13 cases (92%). In this study, not all patients had a same-day comparison with a 12-lead ECG. Marín et al. (24) evaluated the agreement and clinical precision of the KardiaMobile single-lead device (KM-1l). In this study, performed on 128 patients with a confirmed or presumed diagnosis of COVID-19, QTc of ECG recordings obtained with the KM-1l device were compared to QTc obtained with the standard 12-lead ECG. Values of the QTc interval were almost the same for the KM-1l device and the 12-lead ECG (442.45 ± −40.5 vs. 441.65 ± 40.3 ms, *p* **=** 0.15). An excellent agreement and no statistically signiﬁcant differences in the QTc interval measurement was found in this study. It was demonstrated that the KM-1l device has adequate precision and agreement compared to the standard 12-lead ECG. Minquito-Carazo et al. (25) evaluated the feasibility of QTc monitoring with KardiaMobile 6L in 63 COVID-19 patients receiving therapies that could interfere with the QT interval. QTc could be measured in lead II in 84.5% of the registries. In a control group, 12- and 6-lead ECGs were recorded for 45 healthy subjects. It was found that KardiaMobile 6L showed similar diagnostic feasibility for measurement of the QT interval to the standard 12-lead ECG, with moderate reliability. Chung and Guise (26) assessed, in five patients receiving dofetilide for atrial fibrillation, the feasibility of tracings for QTc obtained with the AliveCor device compared to QTc from the standard ECG. No significant difference was found in this study. Pengel et al. (27) compared different devices for ECG monitoring to the standard 12-lead ECG to examine the accuracy of these devices in adults with congenital heart disease. ECG intervals were manually evaluated for these devices. A difference in the QT interval of >40 ms compared to the 12-lead ECG was considered clinically unacceptable. A total of 176 patients were enrolled in this study. In 26%, the QTc difference was >40 ms compared to the standard 12-lead ECG. Azram et al. (28) compared KardiaMobile 6L with the 12-lead ECG in 1,015 unselected cardiac inpatients and outpatients. The QT interval was closely accurate to the gold standard 12-lead ECG. Orchard et al. (29) present data from 30 healthy athletes who underwent a KardiaMobile 6-lead ECG recording and a subsequent 12-lead ECG recording. The difference in the QTc interval was not significant. Koltowski et al. (30) compared KardiaMobile 6L and 12-lead ECGs for a group of 100 consecutive cardiac patients. QT intervals were significantly (*p* < 0.001) shorter in the KardiaMobile 6-lead ECG than in the 12-lead ECG. Beers et al. (31) determined the accuracy of QT measured by KM-1l in 125 patients. These patients had a non-acute indication for a 12-lead ECG. The authors concluded that KM-1l ECGs measured the QT interval accurately compared to standard 12-lead ECGs. Gonzales et al. (32) validated QT intervals measured by KardiaMobile 6L and a conventional ECG in a study on 50 SARS-CoV2 patients. They found a very good correlation between the KardiaMobile 6L device and the 12-lead ECG. The authors showed that the implementing a monitoring protocol can identify patients who are prone to prolong the QT interval and that such devices may represent an alternative for QT interval monitoring. Giudicessi et al. (33) trained and validated an artificial intelligence (AI)-enabled 12-lead ECG algorithm to determine the QTc. They prospectively tested this algorithm on tracings recorded from a mobile ECG device (equivalent to the AliveCor KardiaMobile 6L). A strong agreement appeared between manually evaluated and AI-predicted QTc values (−1.76 ± 23.14 ms). Mayfield et al. (34) described implementing a fully randomized clinical trial with cardiac monitoring. ECG collection was performed with the KardiaMobile 6L device. The authors demonstrated that remote QT interval monitoring can be efﬁciently performed.

### Apple Watch

**Figure 3:**
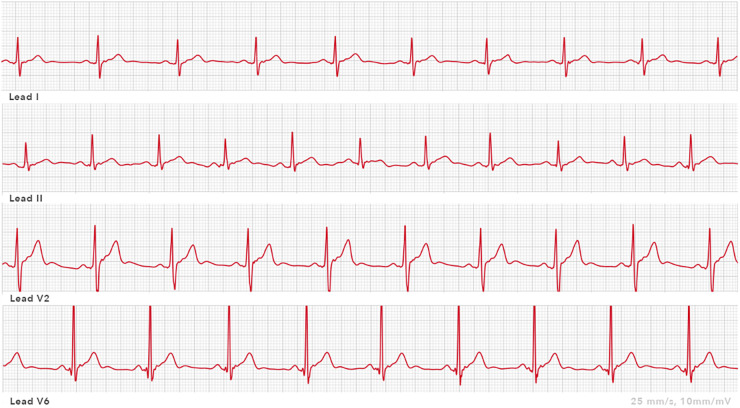
ECG leads recorded by an Apple Watch: lead I Apple Watch ECG 25 mm/s, 10 mm/mV (smartwatch worn on the left wrist); lead II Apple Watch ECG 25 mm/s, 10 mm/mV (smartwatch on left lower abdomen); lead V2 Apple Watch ECG 25 mm/s, 10 mm/mV (smartwatch at the site of V2); and lead V6 Apple Watch ECG 25 mm/s, 10 mm/mV (smartwatch at the site of V6).

Apple Watch Series 3 can record pulse frequency. It uses photoplethysmography located on the back of the watch (35). Apple Watch Series 4 (Apple Inc., Cupertino, CA, USA) has an integrated ECG tool with which a single-lead ECG can be recorded. The negative electrode is placed in the crown, and the positive electrode is located on the back of the watch. A bipolar ECG lead, the simulated lead I, can be derived by recording the voltage difference over time between the watch's back electrode on the left arm wrist and the right index finger on the crown (36, 37). Electrocardiograms can be stored on a smart device mobile application (mApp). Afterward, PDFs can be generated from obtained ECGs. An example of an ECG obtained with an Apple Watch from standard and non-standard positions is shown in Figure 3. This wearable SW contains possibilities to detect atrial fibrillation. Apple Watch has received FDA approval for the detection of atrial fibrillation. Five studies examined the Apple Watch in the context of QT interval measurements. Spaccarotella et al. (38) assessed in 119 patients, admitted to the cardiology division, the feasibility and reliability of the obtained QT interval examined in leads I, II, and V2 using an Apple Watch. Lead I was recorded in the standard SW position with the watch on the left wrist. For leads II and V2, the SW was placed in non-standard positions. Lead II was recorded with the SW on the left lower abdomen; for obtaining lead V2, the SW was placed in the fourth intercostal space left parasternal. For all these above-mentioned leads, the right index finger was placed on the crown. The authors calculated an average of the QT interval in all of the above-mentioned leads (I, II, V2) using Bazett's, Fidericia's, and Framingham's formulas. A strong agreement was found between the QT intervals measured in the different leads compared to standard 12-lead ECGs, so the authors concluded that the Apple Watch can accurately measure the QT interval compared with the standard ECG. Saghir et al. (39) compared the accuracy of interval electrocardiographic interval measurements on Apple Watch ECG tracings to lead 1 on 12-lead ECGs in 43 volunteers. There were no inconclusive readings. Strong agreement, defined as mean difference (d) <20 ms, was found in 65.1% of the QT measurements and 48.8% of the QTc measurements. Moderate agreement, defined as d <40 ms, was found in 86% of the QT intervals and 74.4% of the QTc measurements. Chokshi et al. (40) compared the SW-recorded QT and QTc assessed using AccurKardia's AccurBeat platform with the conventional 12-lead ECG. This study consisted of 50 healthy participants. All analyzable complexes of the 12-lead ECG were in leads I and II. The AccurBeat platform annotates ECGs and can also diagnose arrhythmias using AI-based techniques. More than 90% of the average QT interval measurements between the platform and the QT intervals from the 12-lead ECG were within the 95% CI. The authors concluded that QT and QTc intervals obtained by the Apple SW coupled with the platform are comparable to those from a 12-lead ECG. Strik et al. (41) investigated using the Apple Watch for QT measurement, including using non-standard SW positions, in an unselected outpatient population (*N* = 100). Apple Watch lead I was obtained with the watch on the left wrist, and lead II was obtained with the watch on the left ankle. Furthermore, the simulated lead V6 was recorded with the watch on the left lateral chest. Adequate QT measurements were observed in 85% of the patients when the SW was worn on the left wrist. This number of adequate measurements increased to 94% when the SW was moved to alternative positions. Chinitz et al. (42) published a case report about a physician in home isolation due to a COVID-19 infection. She was prescribed hydroxychloroquine and considered at moderate risk for drug-associated QT prolongation. Recordings from the Apple Watch rhythm strips were transmitted to a cardiologist. After treatment, a 12-lead ECG was performed in the hospital, which showed a very similar waveform morphology and QT measurement to lead I from the Apple Watch.

### Withings Move ECG ScanWatch

The Withings ScanWatch (SW, Withings SA, Issy les Moulineaux, France) is an analog watch with an in-built single-lead ECG. It offers, without manual measurement of the SW-ECG or the need for any other software, an automated analysis of the corrected QT interval (43). An artificial intelligence QTc (AI-QTc) is systematically measured from the smartwatch ECG (SW-ECG). After performing the SW-ECG, it is transmitted for assessment to the Cardiologs platform. The AI-QTc is calculated by a deep convolutional neural network that identified both the onset of QRS complexes and the offset of subsequent T waves in the SW-ECG. Finally, to remove extreme and anomalous values, the AI-QTc of the SW-ECG was calculated as the median QTc over all beats (44). A total of three studies examined this SW. In two studies, the agreement between manual QTc measurement by a 12-lead ECG and the AI-QTc of the SW-ECG was tested. Another study examined the accuracy of different ECG-based devices, including the Withings ScanWatch, compared to the 12-lead standard ECG on several tasks. Mannhart et al. (43) compared automated QTc measurements of the Withings ScanWatch with manually measured QTc from a 12-lead recorded ECG. A total of 317 patients referred for cardiac work-up were enrolled in this study. Two blinded cardiologists manually interpreted the QT interval of a 12-lead ECG by assessing lead II or V5/V6 with Bazett's formula. In 177 patients (56%), the AI algorithm was able to automatically measure the QTc. A 6.6 ms bias [with 95% limit of agreement (LoA) of −59 and 72 ms] was reported comparing manual measurements and QTc calculated by the SW-AI. There was a disagreement between the measurements of <15 ms in 38% of the cases, >20 ms in 54% of the cases, and >30 ms in 29% of the cases. There was a substantial difference, defined as greater than the LoA, between the QTc intervals in 7% of the cases. The authors concluded that this SW-AI algorithm tends to underestimate the QTc interval; furthermore, the use of single-lead SW-ECG for QTc monitoring could be feasible, but further validation is needed. Maille et al. (44) assessed a group of 85 patients with COVID-19. These patients underwent hydroxychloroquine−azithromycin therapy, which is known as a drug that interferes the QT interval. The authors compared the AI-QTc with a manually measured QTc on a 12-lead ECG, measured in leads I and II or V5. This study showed the AI-QTc tends to overestimate QTc compared to the standard 12-lead ECG. At baseline, there was a difference of less than 50 ms between the two measurements in 97% of the patients. On days 6 and 10, there was a difference of less than 50 ms in 96% and 98% of the patients, respectively. The authors concluded that fair agreement was observed between AI and 12-lead ECGs. Pengel et al. (27) compared different devices for ECG monitoring to the standard 12-lead ECG to examine the accuracy of these devices in adults with congenital heart disease. ECG intervals were manually evaluated for these devices. A difference in the QT interval of >40 ms compared to the 12-lead ECG was considered clinically unacceptable. A total of 176 patients were enrolled in this study. In all patients, Withings ScanWatch ECGs were recorded. In 84% of the patients, the QT interval could be assessed and identified. The authors concluded that QTc was underestimated and QTc duration accuracy was acceptable in only 51% of Withings ECGs. In 49%, the QTc difference was >40 ms, assessed by a physician, compared to the 12-lead ECG.

### Other devices

Kaleschke et al. (45) evaluated the diagnostic accuracy of another device (Omron HeartScan HCG-80) in 508 patients with an indication for 12-lead ECG and compared it to that of a standard 12-lead ECG. This study showed a linear correlation of continuous ECG parameters (with also QTc measurement) between Omron HeartScan and the 12-lead ECG in the study population (*R*^2^ = 0.89). Carter et al. (46) evaluated the feasibility and compliance with daily home ECG monitoring of the QT interval in 31 heart transplant patients using the HeartOne (Aerotel Medical Systems, Holon, Israel) device. During the study period, 644 ECGs were successfully received; of these, 569 ECGs (89%) were acceptable for QTc measurement. Bekker et al. (47) assessed the diagnostic accuracy of a single-lead ECG recorder (HeartCheck) for measuring QTc prolongation. The authors concluded an inferior diagnostic accuracy of this device to measure QTc intervals in cardiology patients to the gold-standard 12-lead ECG. Fouassier et al. (48) evaluated the quality of signals measured with a 12-lead acquisition smart T-shirt (Cardioskin) or a 12-lead Holter recording in 30 healthy subjects. All measured parameters, including QTc, were comparable to the ones obtained with the Holter. Garabelli et al. (49) compared QT interval readings between a Smartphone Home Monitor (SHM) and a 12-lead ECG in 99 healthy volunteers and 25 patients receiving sotalol or dofetilide. An AliveCor-designed prototype was used that allowed the recording of various leads. A very good agreement in QT interval measurements was shown between the Smartphone Home Monitor and the 12-lead ECG in healthy volunteers. However, just a reasonable agreement was demonstrated in patients. Castelletti et al. (50) investigated whether automated QTc measurements obtained by BodyGuardian (BG), a wearable remote monitor system, were reliable compared to manual measurements in 20 patients with long QT syndrome and 16 healthy controls. Measurements of the QT interval obtained by BG were very similar to the manual measurements. Nigolian et al. (51) evaluated the diagnostic accuracy of the Beurer ME 80 device (Ulm, Germany) in 52 patients. It was difficult to recognize the waveforms due to technical issues such as lower signal quality and non-simultaneous tracings. Because of this, it was not possible to measure the QT interval, so diagnosis of prolonged QTc was inaccurate. Haverkamp et al. (52) investigated the accuracy and usability of single-lead ECG obtained by ECG Check in 94 cardiac patients admitted to the hospital and 50 asymptomatic controls. No significant differences were found in QT intervals.

## Discussion

Measuring and monitoring QTc intervals are frequently performed in the early phases of novel drug development programs and in daily clinical practice during antiarrhythmic drug initiation. The golden standard for QTc analyses is the 12-lead ECG, but it is not practical to monitor QTc intervals over a longer period of time. During the last few years, many wearable devices that can measure QTc intervals have become available. Only three of them have been adequately compared to 12-lead ECG measurements. Two of these are commercially available smartwatches (Apple Watch and Withings ScanWatch) with possibilities for ECG and QTc measurements. When an SW is worn on the wrist, which is common practice, the device can only provide lead I recording, which has significant limitations. Historically, measurement of conduction intervals is preferably performed in lead II (53), which is not possible when the watch is worn on the wrist. Furthermore, Cheung et al. (54) suggested that the acquisition of accurate and reproducible QTc values is only possible after obtaining multiple leads. However, this limitation can be overcome by performing recording at non-standard positions. This can be done by placing the SW in other places and positions on the body, which improved the accuracy of the Apple Watch from 85% to 94%. The Withings ScanWatch was only studied using a single lead position. The benefit of this Withings ScanWatch is the automated analysis of the corrected QT interval remotely without needing third-party software or manual measurement of SW-ECG. However, this is limited by the finding that the automated algorithm was able to measure QTc in only 56% of cases (43). On the other hand, a fair agreement was found between the QTc interval durations measured manually on a standard 12-lead ECG and assessed by AI on single-lead SW recordings (44). At this time, the Apple Watch does not offer an automated QTc measurement; addition of this feature might be desirable in the future. A cardiology-focused digital health company (AccurKardia) had developed a device diagnostic platform (AccurBeat) to analyze Apple Watch-generated ECGs. It was found that a total of 94% of the average QT interval measurements by the platform and the 12-lead ECG were within the 95% CI of the average difference (40). Some studies have shown that manual measurement is even more accurate (48). However, manual QT interval assessment is time-consuming and tedious, and, even when performed by experts, the discrepancy between manual QTc measurements is wide, ranging from 34 to 80 ms (55). Furthermore, the QT interval is a dynamic parameter due to sympathovagal interaction in diurnal variation (56). The best-studied device was KardiaMobile 6L, an FDA-approved device for QTc analyses in COVID-19 patients that provides a 6-lead ECG. Two studies examined the earlier version of the KMobile-1l device. Most studies found good accuracy between the QTc measurements of the Kardia device and 12-lead ECG. One study found KardiaMobile 6L underestimated the QTc compared to the corresponding 12-lead QTc. However, this was a small study and not all recordings were taken on the same day. In addition to good accuracy, another great advantage of KardiaMobile 6L is that multiple lead recordings were obtained, which improves accuracy. ECG registration time was found to be significantly lower with KardiaMobile 6L compared with the 12-lead ECG, which suggests good usability. A disadvantage of KardiaMobile 6L is that it can only be used to make ECG recordings and offers no other functionalities. Smartwatches offer many functionalities, including the option for ECG recordings. Many households already own an SW, increasing the potential availability of measurements with these devices. Only a single study provided information on their accuracy in measuring QTc intervals from a few other devices. Other studies only described the feasibility and compliance of these devices. Omron HeartScan HCG-801-E, CardioSkin, BodyGuardian, ECG Check, and HeartOne showed comparable QTc results to 12-lead ECGs. The QTc analysis results of Beurer ME 80 and HeartCheck were inferior compared to the 12-lead ECG. QTc measurements by the Smartphone Home Monitor demonstrated very good agreement with the 12-lead ECG in healthy volunteers and reasonable agreement in patients. We note that some of these other investigated devices clearly showed promising results, but hardly anyone had these devices at home, which makes using such a device for monitoring the QTc interval in households less practical. Most studies were performed on patients, either with COVID-19 or various cardiac diseases. Garabelli et al. (49) showed important differences in the accuracy of the same device between patients and healthy volunteers, with very good agreement in healthy volunteers and reasonable agreement in hospitalized patients. This finding suggests that it is recommended for phase 1 studies only to use a device that has also been studied on healthy people. There are clear advantages in monitoring QTc intervals using a smart device. Remote monitoring offers the opportunity to reduce the duration of confinement and might reduce the study burden on the participants as well as the costs of the study. Remote monitoring can also be promising for patients who are prescribed QT-prolonging medications. Another advantage is the potential reduction of the ecological footprint. Because many people already own an SW, no extra material needs to be manufactured for this. Furthermore, less paper is used than if all these ECGs were produced in the traditional way. In addition, less travel, and therefore less CO_2_ emissions, is required because patients have the option of sending an ECG to their doctor from the home. This is an assumption and needs further investigation. However, it can be argued that home measurement of QT intervals may allow for a reduction in time and resources for travel. A potential limitation of using smart devices for measurement of the QT interval is the fact that one of the parts of the Schwartz score, the recommended method for diagnosing prolonged QT intervals, includes measurement of the QT interval after exercise testing (7). Measurement of the QT interval using a smart device after exercise testing has not yet been investigated. Future studies need to focus on several issues. Safety and adequate alerting in case of QT prolongation need to be prospectively studied. Healthy volunteers have been underrepresented in the presented studies. In addition, many studies were conducted during the COVID-19 pandemic. Conducting studies during the COVID-19 pandemic has its limitations, which should be taken into account. Another limitation of the studies comparing 12-lead ECG to SW-ECG is inconsistent criteria for what is considered an acceptable difference between the two measurements. QT intervals, even if corrected for heart rate, are not only prone to change by drug therapy but also by circadian rhythms and vagal and sympathetic tone. This needs to be taken into account when designing future studies. If you think about an optimal situation, a wearable device should be able to transmit ECGs via remote monitoring to the treating physician for periodic QT analysis but also be able to transmit alerts in case of QT prolongation exceeding a certain threshold or in case of proarrhythmic events such as self-limiting TdP.

## Conclusions

Smartwatches and smart devices offer possibilities for monitoring the QT interval and could be of great additional value. Compared to a 12-channel ECG, patients can record an ECG themselves, which is also possible at home. Results differ from device to device, but some devices can provide comparable results with the gold standard 12-lead ECG and allow adequate QT measurements. Given that smartwatches are already owned by many people and offer additional functionalities, these are promising devices. However, it is recommended to not only measure the QT interval from standard lead I but also at least from lead II and preferably one of the precordial leads. Further studies are needed to evaluate and validate QTc monitoring in healthy subjects and patients. While much research has been done into detecting atrial fibrillation with an SW, this review proves that reliable measurement of the QT interval is also possible. This can have an important impact on drug safety monitoring and monitoring of patients at risk for QT prolongation and offers opportunities in drug research. These devices have the potential to lead to future clinical applications in the evaluation of any drug-induced arrhythmogenicity related to prolongation of the QT interval, needing close monitoring of QT intervals. Before they can be used in daily clinical practice for antiarrhythmic drug initiation, alerts for QT prolongation or arrhythmic events need to be prospectively studied.

## References

[1] Jervell A Lange-Nielsen F. Congenital deaf-mutism, functional heart disease with prolongation of the Q-T interval and sudden death. Am Heart J. (1957) 54:59–68. 10.1016/0002-8703(57)90079-0 13435203

[2] Romano C Gemme G Pongiglione R. [Rare cardiac arrythmias of the pediatric age. II. Syncopal attacks due to paroxysmal ventricular fibrillation. (Presentation of 1st case in Italian pediatric literature)]. Clin Pediatr (Bologna). (1963) 45:656–83. Available at: https://pubmed.ncbi.nlm.nih.gov/14158288/ (Accessed February 14, 2023). 14158288

[3] Ward OC. A new familial cardiac syndrome in children. J Ir Med Assoc. (1964) 54:103–6. 14136838

[4] Selzer A Wray HW. Quinidine syncope. Paroxysmal ventricular fibrillation occurring during treatment of chronic atrial arrhythmias. Circulation. (1964) 30:17–26. 10.1161/01.CIR.30.1.17 14197832

[5] Dessertenne F. [Ventricular tachycardia with 2 variable opposing foci]. Arch Mal Coeur Vaiss. (1966) 59:263–72. 4956181

[6] Lankaputhra M Voskoboinik A. Congenital long QT syndrome: a clinician’s guide. Intern Med J. (2021) 51:1999–2011. 10.1111/IMJ.15437 34151491

[7] *Table 1. [Scoring system for clinical diagnosis of long QT syndrome]. GeneReviews®—NCBI Bookshelf*. Available at: https://www.ncbi.nlm.nih.gov/books/NBK1129/table/rws.T.scoring_system_for_clinical_diagno/ (Accessed February 14, 2023).

[8] Meanock CI Noble MIM. A case of prenylamine toxicity showing the torsade de pointes phenomenon in sinus rhythm? Postgrad Med J. (1981) 57:381–4. 10.1136/PGMJ.57.668.381 7301686 PMC2424893

[9] Fung M Thornton A Mybeck K Wu JH-H Hornbuckle K Muniz E. Evaluation of the characteristics of safety withdrawal of prescription drugs from worldwide pharmaceutical markets—1960 to 1999. Drug Inf J. (2001) 35:293–317. 10.1177/009286150103500134

[10] Beach SR Celano CM Sugrue AM Adams C Ackerman MJ Noseworthy PA QT prolongation, Torsades de pointes, and psychotropic medications: a 5-year update. Psychosomatics. (2018) 59:105–22. 10.1016/J.PSYM.2017.10.009 29275963

[11] Shah RR Morganroth J. ICH E14 Q & A (R1) document: perspectives on the updated recommendations on thorough QT studies. Br J Clin Pharmacol. (2013) 75:959–65. 10.1111/J.1365-2125.2012.04442.X 22905923 PMC3612714

[12] Darpo B Ferber G. The new S7B/E14 question and answer draft guidance for industry: contents and commentary. J Clin Pharmacol. (2021) 61:1261–73. 10.1002/JCPH.1880 33896027 PMC9290990

[13] Svennberg E Tjong F Goette A Akoum N di Biase L Bordachar P How to use digital devices to detect and manage arrhythmias: an EHRA practical guide. Europace. (2022) 24:979–1005. 10.1093/EUROPACE/EUAC038 35368065 PMC11636571

[14] Bansal A Joshi R. Portable out-of-hospital electrocardiography: a review of current technologies. J Arrhythm. (2018) 34:129–38. 10.1002/JOA3.12035 29657588 PMC5891427

[15] Serhani MA El Kassabi HT Ismail H Navaz AN. ECG monitoring systems: review, architecture, processes, and key challenges. Sensors (Basel). (2020) 20(6):1796. 10.3390/S20061796 32213969 PMC7147367

[16] Wong KC Klimis H Lowres N Von Huben A Marschner S Chow CK. Diagnostic accuracy of handheld electrocardiogram devices in detecting atrial fibrillation in adults in community versus hospital settings: a systematic review and meta-analysis. Heart. (2020) 106:1211–7. 10.1136/HEARTJNL-2020-316611 32393588

[17] Moher D Liberati A Tetzlaff J Altman DG Group PRISMA. Preferred reporting items for systematic reviews and meta-analyses: the PRISMA statement. Open Med. (2009) 3:e123–30. 21603045 PMC3090117

[18] Bergeman AT Pultoo SNJ Winter MM Somsen GA Tulevski II Wilde AAM Accuracy of mobile 6-lead electrocardiogram device for assessment of QT interval: a prospective validation study. Neth Heart J. (2022) 31(9):340–7. 10.1007/S12471-022-01716-5 36063313 PMC10444736

[19] https://alivecor.com/press/press_release/new-fda-guidance-allows-use-of-kardiamobile-6l-to-measure-qtc-in-covid-19-patients/.

[20] Kleiman R Darpo B Brown R Rudo T Chamoun S Albert DE Comparison of electrocardiograms (ECG) waveforms and centralized ECG measurements between a simple 6-lead mobile ECG device and a standard 12-lead ECG. Ann Noninvasive Electrocardiol. (2021) 26:e12872. 10.1111/ANEC.12872 34288227 PMC8588372

[21] Frisch DR Frankel ES Farzad DJ Woo SH Kubey AA. Initial experience in monitoring QT intervals using a six-lead contactless Mobile electrocardiogram in an inpatient setting. J Innov Card Rhythm Manag. (2021) 12:4433–40. 10.19102/ICRM.2021.120301 33777482 PMC7987428

[22] Abellas-Sequeiros M Lozano-Granero C García-Sebastián C Franco-Díez E Hernández-Madrid A Moreno-Planas J Monitoring of QTc interval in patients with COVID-19. First experience with a portable ECG-recording device. Cardiol J. (2021) 28:483–5. 10.5603/CJ.A2021.0033 33843037 PMC8169181

[23] Puranik S Harlow C Martin L Coleman M Russell G Park M Monitoring prolongation of QT interval in patients with multidrug-resistant tuberculosis and non-tuberculous mycobacterium using mobile health device AliveCor. J Clin Tuberc Other Mycobact Dis. (2021) 26. 10.1016/J.JCTUBE.2021.100293 35146132 PMC8802120

[24] Marín OMM García PÁA Muñoz VOM Castellanos RJC Cáceres ME Santacruz PD. Portable single-lead electrocardiogram device is accurate for QTc evaluation in hospitalized patients. Heart Rhythm O2. (2021) 2:382–7. 10.1016/J.HROO.2021.06.005 34223287 PMC8237373

[25] Minguito-Carazo C Echarte-Morales J Benito-González T del Castillo-García S Rodríguez-Santamarta M Sánchez-Muñoz E. QT interval monitoring with handheld heart rhythm ECG device in COVID-19 patients. Glob Heart. (2021) 16(1):42. 10.5334/GH.916 34211828 PMC8195254

[26] Chung EH Guise KD. QTC intervals can be assessed with the AliveCor heart monitor in patients on dofetilide for atrial fibrillation. J Electrocardiol. (2015) 48:8–9. 10.1016/J.JELECTROCARD.2014.10.005 25453194

[27] Pengel LKD Robbers-Visser D Groenink M Winter MM Schuuring MJ Bouma BJ A comparison of ECG-based home monitoring devices in adults with CHD. Cardiol Young. (2023) 33(7):1129–35. 10.1017/S1047951122002244 35844104

[28] Azram M Ahmed N Leese L Brigham M Bowes R Wheatcroft SB Clinical validation and evaluation of a novel six-lead handheld electrocardiogram recorder compared to the 12-lead electrocardiogram in unselected cardiology patients (EVALECG cardio). Eur Heart J Digit Health. (2021) 2:643–8. 10.1093/EHJDH/ZTAB083 36713105 PMC9707882

[29] Orchard JJ Orchard JW Raju H la Gerche A Puranik R Semsarian C. Comparison between a 6-lead smartphone ECG and 12-lead ECG in athletes. J Electrocardiol. (2021) 66:95–7. 10.1016/J.JELECTROCARD.2021.03.008 33878565

[30] Koltowski L Balsam P Glowczynska R Rokicki JK Peller M Maksym J Kardia Mobile applicability in clinical practice: a comparison of Kardia Mobile and standard 12-lead electrocardiogram records in 100 consecutive patients of a tertiary cardiovascular care center. Cardiol J. (2021) 28:543–8. 10.5603/CJ.A2019.0001 30644079 PMC8276994

[31] Beers L van Adrichem LP Himmelreich JCL Karregat EPM de Jong JSSG Postema PG Manual QT interval measurement with a smartphone-operated single-lead ECG versus 12-lead ECG: a within-patient diagnostic validation study in primary care. BMJ Open. (2021) 11. 10.1136/BMJOPEN-2021-055072 34732504 PMC8572408

[32] González NT Acosta LÁ Miranda DV Plasencia AI Cáceres VB Zambrano MR QT interval measurement with portable device during COVID-19 outbreak. Int J Cardiol Heart Vasc. (2020) 30:100644. 10.1016/J.IJCHA.2020.100644 32964098 PMC7498206

[33] Giudicessi JR Schram M Bos JM Galloway CD Shreibati JB Johnson PW Artificial intelligence-enabled assessment of the heart rate corrected QT interval using a mobile electrocardiogram device. Circulation. (2021) 143:1274–86. 10.1161/CIRCULATIONAHA.120.050231 33517677

[34] Mayfield JJ Chatterjee NA Noseworthy PA Poole JE Ackerman MJ Stewart J Implementation of a fully remote randomized clinical trial with cardiac monitoring. Commun Med. (2021) 1:62. 10.1038/S43856-021-00052-W 35604806 PMC9053200

[35] Foster KR Torous J. The opportunity and obstacles for smartwatches and wearable sensors. IEEE Pulse. (2019) 10:22–5. 10.1109/MPULS.2018.2885832 30872210

[36] Samol A Bischoff K Luani B Pascut D Wiemer M Kaese S. Recording of bipolar multichannel ECGs by a smartwatch: modern ECG diagnostic 100 years after einthoven. Sensors (Basel). (2019) 19(13):2894. 10.3390/S19132894 31261981 PMC6651039

[37] Avila CO. Novel use of Apple Watch 4 to obtain 3-lead electrocardiogram and detect cardiac ischemia. Perm J. (2019) 23. 10.7812/TPP/19-025 31314734 PMC6636475

[38] Spaccarotella CAM Migliarino S Mongiardo A Sabatino J Santarpia G de Rosa S Measurement of the QT interval using the Apple Watch. Sci Rep. (2021) 11:10817. 10.1038/S41598-021-89199-Z 34031432 PMC8144193

[39] Saghir N Aggarwal A Soneji N Valencia V Rodgers G Kurian T. A comparison of manual electrocardiographic interval and waveform analysis in lead 1 of 12-lead ECG and Apple Watch ECG: a validation study. Cardiovasc Digit Health J. (2020) 1:30–6. 10.1016/J.CVDHJ.2020.07.002 35265871 PMC8890353

[40] Chokshi S Tologonova G Calixte R Yadav V Razvi N Lazar J Comparison between QT and corrected QT interval assessment by an Apple Watch with the AccurBeat platform and by a 12-lead electrocardiogram with manual annotation: prospective observational study. JMIR Form Res. (2022) 6:e41241. 10.2196/41241 36169999 PMC9557757

[41] Strik M Caillol T Ramirez FD Abu-Alrub S Marchand H Welte N Validating QT-interval measurement using the Apple Watch ECG to enable remote monitoring during the COVID-19 pandemic. Circulation. (2020) 142:416–8. 10.1161/CIRCULATIONAHA.120.048253 32478565 PMC7382529

[42] Chinitz JS Goyal R Morales DC Harding M Selim S Epstein LM. Use of a smartwatch for assessment of the QT interval in outpatients with coronavirus disease 2019. J Innov Card Rhythm Manag. (2020) 11:4219–22. 10.19102/ICRM.2020.1100904 32983590 PMC7510469

[43] Mannhart D Hennings E Lischer M Vernier C de Lavallaz JdF Knecht S Clinical validation of automated corrected QT-interval measurements from a single lead electrocardiogram using a novel smartwatch. Front Cardiovasc Med. (2022) 9. 10.3389/FCVM.2022.906079 35811720 PMC9259864

[44] Maille B Wilkin M Million M Rességuier N Franceschi F Koutbi-Franceschi L Smartwatch electrocardiogram and artificial intelligence for assessing cardiac-rhythm safety of drug therapy in the COVID-19 pandemic. The QT-logs study. Int J Cardiol. (2021) 331:333–9. 10.1016/J.IJCARD.2021.01.002 33524462 PMC7845555

[45] Kaleschke G Hoffmann B Drewitz I Steinbeck G Naebauer M Goette A Prospective, multicentre validation of a simple, patient-operated electrocardiographic system for the detection of arrhythmias and electrocardiographic changes. Europace. (2009) 11:1362–8. 10.1093/EUROPACE/EUP262 19797150

[46] Carter EV Hickey KT Pickham DM Doering LV Chen B Harris PRE Feasibility and compliance with daily home electrocardiogram monitoring of the QT interval in heart transplant recipients. Heart Lung. (2012) 41:368–73. 10.1016/J.HRTLNG.2012.02.012 22459508 PMC3387335

[47] Bekker CL Noordergraaf F Teerenstra S Pop G van den Bemt BJF. Diagnostic accuracy of a single-lead portable ECG device for measuring QTc prolongation. Ann Noninvasive Electrocardiol. (2020) 25(1):e12683. 10.1111/ANEC.12683 31350811 PMC7050507

[48] Fouassier D Roy X Blanchard A Hulot JS. Assessment of signal quality measured with a smart 12-lead ECG acquisition T-shirt. Ann Noninvasive Electrocardiol. (2020) 25(1):e12682. 10.1111/ANEC.12682 31339208 PMC7050501

[49] Garabelli P Stavrakis S Albert M Koomson E Parwani P Chohan J Comparison of QT interval readings in normal sinus rhythm between a smartphone heart monitor and a 12-lead ECG for healthy volunteers and inpatients receiving sotalol or dofetilide. J Cardiovasc Electrophysiol. (2016) 27:827–32. 10.1111/JCE.12976 27027653

[50] Castelletti S Dagradi F Goulene K Danza AI Baldi E Stramba-Badiale M A wearable remote monitoring system for the identification of subjects with a prolonged QT interval or at risk for drug-induced long QT syndrome. Int J Cardiol. (2018) 266:89–94. 10.1016/J.IJCARD.2018.03.097 29887480

[51] Nigolian A Dayal N Nigolian H Stettler C Burri H. Diagnostic accuracy of multi-lead ECGs obtained using a pocket-sized bipolar handheld event recorder. J Electrocardiol. (2018) 51:278–81. 10.1016/J.JELECTROCARD.2017.11.004 29223306

[52] Haverkamp HT Fosse SO Schuster P. Accuracy and usability of single-lead ECG from smartphones—a clinical study. Indian Pacing Electrophysiol J. (2019) 19:145–9. 10.1016/J.IPEJ.2019.02.006 30794928 PMC6697525

[53] Postema P Wilde A. The measurement of the QT interval. Curr Cardiol Rev. (2014) 10:287–94. 10.2174/1573403X10666140514103612 24827793 PMC4040880

[54] Cheung CC Davies B Gibbs K Laksman ZW Krahn AD. Multilead QT screening is necessary for QT measurement: implications for management of patients in the COVID-19 era. JACC Clin Electrophysiol. (2020) 6:878–80. 10.1016/J.JACEP.2020.04.001 32703574 PMC7141442

[55] Viskin S Rosovski U Sands AJ Chen E Kistler PM Kalman JM Inaccurate electrocardiographic interpretation of long QT: the majority of physicians cannot recognize a long QT when they see one. Heart Rhythm. (2005) 2:569–74. 10.1016/J.HRTHM.2005.02.011 15922261

[56] Murakawa Y Inoue H Nozaki A Sugimoto T. Role of sympathovagal interaction in diurnal variation of QT interval. Am J Cardiol. (1992) 69:339–43. 10.1016/0002-9149(92)90230-V 1734645

